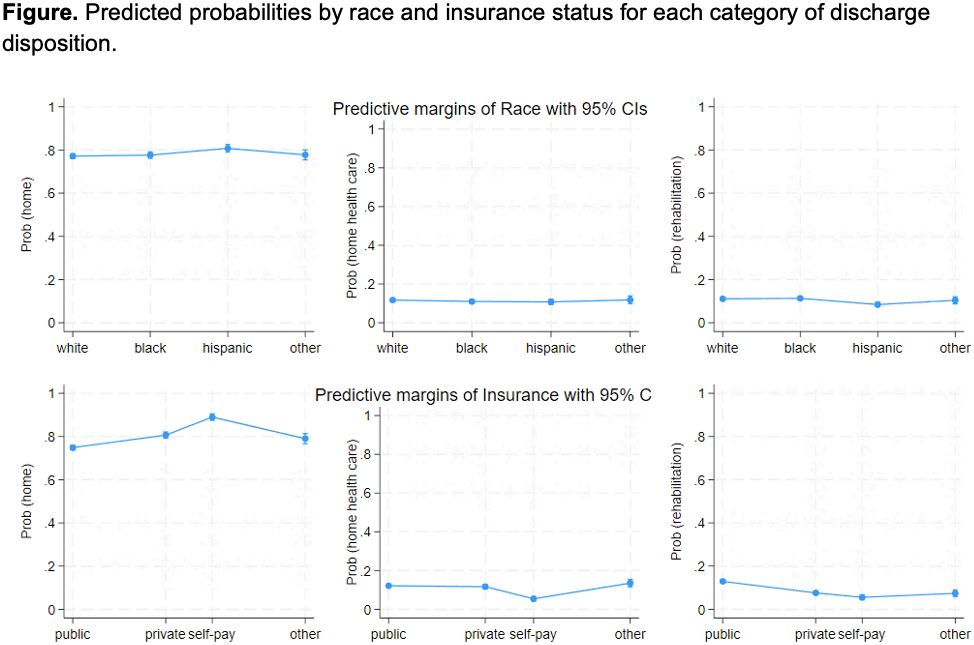# 906 Racial and Socioeconomic Disparities in Discharge Disposition of Burn Survivors

**DOI:** 10.1093/jbcr/iraf019.437

**Published:** 2025-04-01

**Authors:** Manuel Castillo-Angeles, Katie Foley, Barbara Okafor, Anupama Mehta

**Affiliations:** Brigham and Women’s Hospital; Brigham and Women’s Hospital; Brigham and Women’s Hospital; Brigham and Women’s Hospital

## Abstract

**Introduction:**

After hospitalization, rehabilitation is an essential component of recovery for burn injury survivors. However, access to it after discharge depends on multiple factors. It is well established that racial and socio-demographic disparities exist in healthcare, frequently resulting in unfavorable outcomes for these subpopulations. The aim of this study was to assess the association between racial and socioeconomic disparities and discharge disposition, and subsequent access to rehabilitation, after burn injury.

**Methods:**

This is a retrospective study using the National Inpatient Sample (NIS) 2015-2018. All adult inpatients with a primary diagnosis of burn injury were included. Our main exposure variables included race (White, Black, Hispanic, Other), insurance type (expected primary payer: Private, public, self-pay/uninsured, other) and median household income for patient’s ZIP Code (as a marker for socio-economic status). Our main outcome was discharge disposition, categorized into home without support, home health care, and rehabilitation (included Skilled Nursing Facility, Intermediate Care Facility, inpatient rehabilitation facility). Multinomial logistic regression was used to identify the association between socio-demographic factors and our main outcome.

**Results:**

A total of 138,095 weighted burn injury admissions were identified. 15,190 (11%) were discharged to rehabilitation. In adjusted analysis, Hispanic patients had lower probability of discharge to home health care (Relative Risk Ratio [RRR] 0.83, 95% Confidence Interval [CI] 0.70 – 0.99) or rehabilitation (RRR 0.67, 95%CI 0.56–0.81) vs. being discharged home compared with White patients. Compared with private insurance, uninsured patients had lower probability of discharge to rehabilitation (RRR 0.59, 95%CI 0.45–0.77), but having public insurance was associated with an increased probability of discharge to rehabilitation (RRR 2.01, 95%CI 1.75–2.29) vs. being discharged home. Median household income for patient’s ZIP Code was not associated with our outcome. The figure shows predicted probabilities by race and insurance status for each category of discharge disposition.

**Conclusions:**

Racial and socio-economic disparities persist in discharge destination among burn survivors and thus, access to rehabilitation services. Further studies need to focus on developing and implementing targeted interventions to ensure equality in the access to better care after burn injury.

**Applicability of Research to Practice:**

Understanding the implications of these disparities on access to rehabilitation services may help clinicians better tailor care and resources to optimize survivor recovery.

**Funding for the Study:**

N/A